# 

**Published:** 1872-01

**Authors:** 


### BIBLIOGRAPHICAL.

---

*Transactions of the Southern Dental Association*, 1871.—A record of the proceedings of the third annual meeting, held at Charleston, S. C., April 12th to 15th, inclusive, handsomely gotten up in a volume of 63 pages, and in a style superior to any heretofore published.

*Physical Diagnosis of Brain Disease.*—By Reuben A. Vance, M. D. Reprinted from the "Medical World" in pamphlet form.

*Clinical Examination of Urine.*—With a description of a convenient apparatus for its speedy analysis. By Reuben A. Vance, M. D. Reprinted from "Medical World" in pamphlet form of twelve pages. Wm. Baldwin, Publisher, New York.

*Syphilitic Epilepsy.*—By Reuben A. Vance, M. D. Reprinted from the "Amer. Jour. of Syphilography and Dermatology," in pamphlet form of fifteen pages. F. W. Christian, Publisher, New York.

*Sugar Formation in the Liver.*—By J. C. Dalton, M. D. Reprinted in pamphlet form from the "Transactions of the New York Academy of Medicine." Illustrated.

*Transactions of the Am. Ophthalmological Society.* Eighth annual meeting held at Newport, July, 1871. D. Appleton & Co. Publishers; containing a number of valuable papers and illustrations, neatly arranged in a pamphlet of 145 pages.

*A Contribution to the Treatment of the Versions and Flexions of the Unimpregnated Uterus.*—By Ephraim Cutler, A. M., M. D. Reprinted from "Jour. of the Gynaecological Society." James Campbell, Publisher, Boston. Illustrated by twenty wood cuts.

*The American Agriculturist.* For the Farm Garden and Household. Published by Orange Judd & Co., 243 Broadway, New York. The January number of this valuable monthly, commences the thirty-first volume, and is a specimen of what the energetic publishers have furnished to their subscribers for many years past. It has no superior in the agricultural literature of the day. Published in both English and German at $1.50 per annum. Single number 15 cts.

*The Prevention of Abscesses in Hyperdermic Medication.*—By Reuben A. Vance, M. D. W. Baldwin & Co., Publishers, New York. Reprinted in pamphlet form from the "Medical World."

*The Mutual Relations of the Medical Profession. Its Press and the Community.* By Dr. Storer, Jr. (Horatio.) Published by James Campbell, Boston. An able address delivered at the annual meeting of Association of Medical Editors, on May 1st, 1871, at San Francisco, Cal.

*Medical Education in America.* By Henry J. Bigelow, M. D. of Harvard University. An excellent address read before the Mass. Medical Society, June 7th, 1871. Published by Welch, Bigelow, & Co., University Press, Cambridge.

*Vick's Illustrated Catalogue and Floral Guide.* James Vick, Rochester, N. Y., Contains over one hundred pages, printed on tinted paper, with four hundred engravings of flowers, plants and vegetables. The two colored plates are superb, and the present number excels even that of last year. Mr. Vick certainly merits all the honor he is receiving both at home and abroad.

---

